# Association of parental holding during therapeutic hypothermia and NICU outcomes for infants with hypoxic-ischemic encephalopathy

**DOI:** 10.1038/s41372-026-02753-3

**Published:** 2026-06-22

**Authors:** Tiana T. Nguyen, Hannah C. Glass, Natalie Chan, Emily Taketa, Roberta Pineda, Marie-Coralie Cornet, Matthew J. Miller

**Affiliations:** 1 Department of Physical Therapy and Rehabilitation Sciences, University of California, San Francisco, CA, USA.; 2 Department of Neurology and Weill Institute for Neuroscience, Department of Pediatrics and Benioff Children’s Hospital, Department of Epidemiology & Biostatistics, University of California, San Francisco, CA, USA.; 3 Department of Pediatrics and Benioff Children’s Hospital, University of California, San Francisco, CA, USA.; 4 School of Medicine, University of California San Francisco, San Francisco, CA, USA.; 5 Chan Division of Occupational Science and Occupational Therapy, University of Southern California, Los Angeles, CA, USA.

## Abstract

**OBJECTIVE:**

To examine associations between parental holding during therapeutic hypothermia (TH) and neonatal intensive care unit (NICU) outcomes among infants with hypoxic-ischemic encephalopathy (HIE).

**STUDY DESIGN:**

Retrospective cohort of 379 infants with HIE at two level IV NICUs (2017–2024). Unadjusted and adjusted regression models evaluated associations between holding during TH and NICU outcomes.

**RESULT:**

Overall, 28% of infants were held during TH. Holding was less common among infants with greater clinical severity, public insurance, or those identified as Hispanic, Asian, or Black. After adjustment for clinical and sociodemographic factors, holding was associated with faster time to full oral feeding (HR = 1.4, *p* < 0.01), earlier age at full oral feeding (HR = 1.6, *p* < 0.01), shorter hospitalization (*β* = −7.5, *p* = 0.02), and higher odds of breastmilk feeding at discharge (OR = 3.2, *p* = 0.03).

**CONCLUSION:**

Parental holding during TH is associated with favorable short-term NICU outcomes, supporting its potential role as a family-centered care practice during neonatal neurocritical care.

## BACKGROUND

Therapeutic hypothermia (TH) is the standard of care for neonates with moderate to severe hypoxic-ischemic encephalopathy (HIE), a condition caused by reduced oxygenated blood flow to the brain around the time of birth [1]. TH is initiated within the first six hours of birth and involves placing infants on a cooling blanket to maintain their core body temperature at 33–34 °C for 72 h [1, 2]. TH has been shown to significantly reduce mortality and severe neurodevelopmental morbidity among infants with HIE [2].

During TH, infants require admission to a neonatal intensive care unit (NICU) for cardiovascular and neuromonitoring, temperature management, parenteral nutrition, respiratory support, often with invasive ventilation and sedation. The separation between infants and parents, shortly after birth, limits opportunities for early parental engagement during a critical period of neurodevelopment [3]. Although TH has improved survival and neurodevelopmental outcomes, up to 44% of treated infants continue to have substantial neurodevelopmental impairments, emphasizing the need for adjunctive, family-centered interventions to improve long-term health and developmental outcomes [2, 4, 5].

Parental holding, including skin-to-skin contact, in the NICU has been shown to support physiologic stability, feeding outcomes, and neurobehavioral outcomes, and reduce infant stress and hospital length of stay [6–11]. While extensive evidence supports the use of skin-to-skin holding for preterm and term NICU populations, with earlier initiation and greater frequency yielding greater benefits [8, 10]. There is limited research evaluating holding practices during TH.

Traditional skin-to-skin holding is not feasible during TH due to concerns about raising core temperature [12]. However, modified forms of parental holding, such as holding with a cooling blanket in place, have been described [13]. Emerging studies suggest this modified holding is feasible and safe, with no increase in adverse outcomes such as temperature instability or equipment dislodgment [12–15]. One study also reported a reduction in salivary cortisol levels, suggesting potential stress-reducing benefits for both infants and mothers [11]. Despite this growing evidence, implementation varies widely across NICUs, and little is known about the clinical factors that influence whether infants are held during TH [16]. Furthermore, the impact of holding during TH on short-term clinical outcomes has not been well characterized. Therefore, the objectives of this study were to [1] identify infant factors associated with parental holding during TH, and [2] examine the relationship between holding during TH and key NICU outcomes, including time to full oral feeding, hospital length of stay, and breastmilk feeding at discharge.

## METHODS

### Study design and population

We conducted a retrospective cohort study of infants diagnosed with HIE who were admitted to the University of California, San Francisco (UCSF) Benioff Children’s Hospital (BCH) San Francisco campus for TH between March 2017 and February 2024, and the Oakland campus between March 2020 and February 2024, and who completed the full 72 h of TH. Data from the Oakland campus was not available in the database before March 2020. Infants who died during their birth hospitalization and those transferred to another NICU were excluded, as the primary outcomes were not available for these infants.

### Study setting

Both NICUs are Level IV NICUs and regional referral centers serving socioeconomically and racially diverse populations across Northern California. They provide specialized care including TH for critically ill neonates. The San Francisco NICU is co-located within a birthing hospital and receives both inborn and outborn infants. The Oakland NICU only admits outborn infants.

TH was delivered per institutional protocols using commercially available servo-controlled devices (Blanketrol II or Arctic Sun) to maintain a target core temperature of 33–34 °C for 72 h, followed by controlled rewarming. Standard care included continuous video-electroencephalogram monitoring, invasive or non-invasive respiratory support as clinically indicated, and continuous sedation with morphine and/or dexmedetomidine. Central lines (umbilical venous and arterial lines) were routinely placed for vascular access and physiological monitoring. During the study period, there was no standardized holding guideline in place. Instead, decisions regarding parental holding during TH were determined on a case-by-case basis. Modified holding during TH refers to parental holding while the infant remained on the cooling blanket, supported by a pillow between the infant and caregiver, without interrupting temperature targets or device function.

### Measures

All data were extracted from the electronic medical record. The primary variable of interest was whether the infant was held during TH. Other measures included the infant age in days at first hold and the frequency of holding (average number of documented holds per day). Nurses recorded each time the infant was held in the electronic medical record as part of their care.

Comprehensive infant characteristics and clinical factors included gestational age at birth; birth weight; sex assigned at birth (female or male); race and ethnicity as reported by parent (White, Hispanic, Asian, Black, multiracial, or other); insurance type (private or public); delivery type (vaginal or Cesarean section); Apgar scores at 1 and 5 min; severity of encephalopathy based on the modified Sarnat examination between 1 and 6 h after birth; arterial cord pH; arterial cord base excess; brain magnetic resonance imaging (MRI) results indicating basal ganglia, thalamus, or brainstem injury; history of seizures; type of continuous sedation medication used during TH (morphine, dexmedetomidine, both, or none); number of as needed (PRN) morphine doses during TH; ever intubated during TH; total number of intubation days during TH; and whether the infant received a gastrostomy tube placement. Encephalopathy severity was determined using the Sarnat staging systems [4] by a single blinded reviewer. When explicit scores were unavailable, stages were inferred from descriptive neurologic findings or the neonatologist’s documented assessment.

NICU outcomes included time to full oral feeding, day of life at full oral feeding, receipt of breastmilk at discharge, and length of stay. Time to full oral feeding was defined as the number of days from initiation of oral feeding to attainment of full oral feeding. The day of life at full oral feeding was defined as the number of days from birth to attainment of full oral feeding. Full oral feeding was defined as exclusive oral intake without the need for nutritional supplementation via nasogastric tube feeding, parenteral nutrition, or intravenous fluids during hospitalization.

### Data analysis

A manual chart review of key variables (e.g., modified Sarnat score) was conducted to reconcile documentation differences across sites and verify data accuracy and completeness. Data analysis was performed using R (4.4.2). Descriptive statistics were used to define frequencies and central tendencies of infant characteristics, holding measures, and NICU outcomes. Pearson’s chi-square, independent t-tests, and Wilcoxon rank-sum tests were used to compare differences in infant characteristics, time to first hold, frequency of holding, time to full oral feeding, day of life at full oral feeding, whether the infant received breastmilk at discharge, and length of stay between infants held and not held during TH.

Regression models were used to examine the associations between holding during TH and NICU outcomes (time to full oral feeding, day of life at full oral feeding, whether the infant received breastmilk at discharge, and length of stay). Time to full oral feeding and day of life at full oral feeding were analyzed using Cox proportional hazards regression. Infants who underwent gastrostomy tube placement prior to achieving full oral feeding were censored at the time of gastrostomy tube placement, and those who remained hospitalized without achieving full oral feeding after 60 days of life were censored at 60 days to limit the influence of unrelated medical complications or other factors not directly associated with feeding progression. Because gastrostomy tube placement precludes attainment of full oral feeding during the index hospitalization, Fine–Gray subdistribution hazards models treating gastrostomy tube placement as a competing event were performed as a sensitivity analysis and yielded similar results. Kaplan–Meier survival curves were generated to visualize time to full oral feeding and day of life at full oral feeding by parental holding during TH, and the log-rank test was used to compare survival distributions between groups. Results from Cox models are presented as Hazard ratios with 95% confidence intervals (CIs). Breastmilk feeding at discharge was analyzed using logistic regression, with results reported as odds ratios with 95% CIs. Length of stay was analyzed using linear regression, with results presented as β coefficients (mean differences in days) with 95% CIs.

Unadjusted and adjusted models were estimated for each outcome. Adjusted models accounted for severity of encephalopathy, the number of intubation days, race/ethnicity, insurance type, admission year, and site. Covariate selection was guided by a conceptual causal framework (Suppl. Fig. 1). Clinical factors, including severity of encephalopathy and number of intubation days, as well as site and admission year, were considered potential confounders of the relationship between holding and NICU outcomes. Race/ethnicity and insurance type were conceptualized as proxies for structural and institutional factors influencing access to holding and were therefore examined as predictors of exposure. To evaluate the potential for overadjustment, sensitivity analyses were performed excluding race/ethnicity and insurance from outcome models. All tests were two-sided, and statistical significance was defined as *p* < 0.05.

## RESULTS

A total of 440 infants with HIE received TH at the study sites during the study period. Sixty-one infants were excluded (21 died, 40 transferred), resulting in a final analytic sample of 379 infants, with a mean gestational age of 39.4 weeks and a mean birth weight of 3.3 kg (Table 1). Of the 379 infants, 107 (28%) were held during TH. The proportion of infants held during TH increased over time, particularly in later years of the study period, with variation observed across sites (Fig. 1). Among infants held during TH, the time to first hold (median [IQR]) was 1.4 days (0.9–2.1) compared with 4.0 days (3.6–4.9) for infants not held during TH. In addition, infants held during TH had a higher daily frequency of holding during their hospitalization compared to those that were not held during TH (3.0 [2.4–3.9] vs. 2.5 [1.8–3.2], *p* < 0.01).

### Characteristics associated with infant holding during therapeutic hypothermia

Infant holding during TH was more common among infants with higher 5 min Apgar scores (5 [3–7] vs. 4 [3–6], *p*= 0.03), no intubation (47% vs. 17%, *p* < 0.01), private insurance (68% vs. 32%, *p* < 0.01), and admission at Site 2 (34% vs. 23%, *p*= 0.03). Race and ethnicity were significantly associated with holding (*p*= 0.01), with higher rates among infants identified as White or Multiracial and lower rates among those identified as Hispanic, Asian, or Black. No associations were observed between holding during TH and gestational age, birth weight, sex, pH, base excess, presence of brain injury on MRI, or seizures.

### Holding during therapeutic hypothermia and NICU outcomes

Infants who were held during TH experienced more favorable NICU outcomes compared with those who were not held. Infants held during TH achieved full oral feeding earlier (median 2.6 days [1.4–5.2] vs. 3.6 days [1.8–7.7], *p*= 0.04), were more likely to receive breastmilk at discharge (92% vs 78%, *p*= .03), and had a shorter hospital length of stay (median 8 days [7–12] vs. 11 days [8–20], *p* < 0.01) (Table 2).

Unadjusted time-to-event analyses accounting for censoring demonstrated that infants who were held during TH achieved full oral feeding earlier than those not held, whether measured from birth (day of life at full oral feeding) or from initiation of oral feeding (time to full oral feeding) (Fig. 2). For the time to full oral feeding, the unadjusted median time was 2.6 days (95% CI 2.2–3.5) among held infants and 3.8 days (95% CI 3.1–4.7) among those not held. When measured from birth, the median day of life at full oral feeding was 5.9 days (95% CI 5.1–6.7) among held infants and 8.4 days (95% CI 7.7–9.9) among those not held.

All adjusted analyses controlled for severity of encephalopathy, number of intubation days, insurance type, race/ethnicity, admission year, and site. In adjusted analyses, holding during TH was associated with a faster rate of achieving full oral feeding from initiation of oral feeding (HR 1.4, 95% CI 1.1–1.9, *p* < 0.01) and from birth (HR 1.6, 95% CI 1.2–2.0, *p* < 0.01). Infants held during TH were also 2.5 times more likely to receive breastmilk at discharge (95% CI: 1.1–6.1, *p*= 0.03) and had an average of 4.9 days shorter hospital length of stay (95% CI −9.1 to −0.07, *p*= 0.02) than infants not held during TH. In sensitivity analyses excluding race/ethnicity and insurance from adjusted models, results were consistent with the primary analyses, with all associations remaining statistically significant (Suppl. Table 1).

## DISCUSSION

In this two-site retrospective cohort study of infants with HIE, parental holding during TH was independently associated with more favorable NICU outcomes, including earlier attainment of full oral feeding, shorter hospital length of stay, and greater likelihood of breastmilk feeding at discharge. These associations persisted after adjusting for key clinical and contextual factors, suggesting that holding during TH may be an important component of family-centered care for infants receiving neurocritical treatment. In our cohort, approximately 28% of infants were held during TH, highlighting an opportunity for broader implementation.

Our findings revealed variability in holding practices based on clinical, sociodemographic, and institutional factors. Clinically, infants with lower 5 min Apgar scores and intubation were less likely to be held during TH, whereas early diagnostic markers used to guide initiation of TH (pH, base excess, and encephalopathy severity) and seizure occurrence were not significantly associated with holding. Collectively, these findings suggest that decisions about holding during TH may be shaped more by perceived procedural risk and ongoing device burden (i.e., airway management and the complexity of safely transferring an infant with multiple supports) than by early diagnostic severity once TH has been initiated.

In addition to clinical factors, differences in holding practices were observed across sociodemographic groups. Infants who identified as Hispanic, Asian, or Black, as well as those with public insurance, were less likely to be held during TH. These differences likely reflect structural and institutional factors within care delivery systems, including variation in access, communication, and support for family presence, as well as the potential influence of implicit bias, rather than differences in family engagement alone. Prior work has demonstrated inequities in family-centered care practices in NICUs, shaped by systemic barriers such as transportation, childcare constraints, and inequitable parental leave, as well as differences in how care is offered and facilitated across populations [17–21]. Addressing these disparities will require intentional, equity-focused strategies to ensure that all families have consistent opportunities to participate in holding during TH.

Consistent with prior literature, our results suggest that holding during TH can be performed in select critically ill neonates requiring intensive interventions such as intubation, sedation, and seizure management [12–15]. Among infants who were held, holding typically began after the first day of life, corresponding to initiation after the first 24 h of TH. The initial 24 hours of TH often represent a period of heightened physiological instability as infants adjust to TH, sedation regimens, and respiratory support. Clinicians appropriately prioritize stabilization, including sedation titration, initiation or adjustment of ventilatory support, and close monitoring for seizures or hemodynamic fluctuations, during this critical early window. Although intubated infants were less likely to be held, a subset were successfully held during TH, demonstrating that holding can be performed even in higher-acuity patients when appropriate staffing, training, and expertise are available. This aligns with prior work describing provider safety concerns (e.g., risk of unplanned extubation), institutional challenges (e.g., limited staff training), and infant-specific instability as barriers to holding and skin-to-skin care in NICU populations [22, 23]. Addressing these challenges through standardized protocols, staff education, and interdisciplinary support may help expand holding opportunities for infants undergoing TH.

Several mechanisms may explain the observed associations between holding during TH and improved NICU outcomes. Although traditional skin-to-skin care involves direct skin contact and is typically not feasible during TH due to concerns about temperature management, physical closeness during modified holding may preserve many of its sensory and physiologic benefits, including oxytocin release, reduced stress, improved physiological regulation, and neural connectivity [6, 8, 9, 24, 25]. Despite the absence of direct skin contact, holding has been associated with lower salivary cortisol levels in both infants and caregivers, supporting stress reduction and physiological stability, which may facilitate feeding progression and readiness for discharge [26]. Additionally, holding may promote caregiver confidence and engagement, alleviate anxiety and depression, and strengthen caregiver-infant bonding, all of which may indirectly improve NICU outcomes [6–8, 11, 15, 27]. Enhanced bonding and neuroendocrine stimulation may also support lactation and the sustained provision of breastmilk [6, 8, 28]. The observation of these associations in the context of modified holding, rather than traditional skin-to-skin care, suggests that meaningful benefits may be achieved even when direct skin-to-skin contact is not possible. The broader psychosocial benefits of holding, such as reduced caregiver stress and improved parent-infant relationships, may have long-term implications for neurodevelopmental trajectories and should be incorporated into future studies.

We also observed differences in holding practices between hospital sites. Variations may stem from differences in unit policies, clinical workflows, staff training, or patient populations. Similar institutional variation has been reported in skin-to-skin care practices among preterm infants, where implementation is often influenced by factors such as staffing constraints, concerns about medical stability, lack of standardized protocols, and variation in caregiver engagement and provider beliefs about safety and feasibility [23]. These studies suggest that even within high-level NICUs, the presence or absence of supportive infrastructure and unit culture can significantly impact whether skin-to-skin is offered equitably. At the end of the study period, one site began implementing a formal protocol to support parental holding during TH. As this likely involved a gradual transition in practice, infants cared for during this time may have been exposed to evolving approaches to holding. This may have contributed to observed differences in holding practices across sites. The parallel between these findings and our results highlights the need for targeted implementation strategies that address site-specific strategies and promote consistent opportunities for holding during TH. Future research should examine contextual factors that influence clinician decision-making to support the implementation of family-centered care practices more reliably across institutions. Differences in cooling equipment and sedation practices across sites may also contribute to variation in holding practices and warrant further investigation.

While skin-to-skin care is well-studied in preterm populations, holding during TH for infants with HIE remains understudied. This study contributes to the growing body of evidence supporting family engagement in neonatal neurocritical care. Holding during TH is a potentially modifiable practice that can be implemented with appropriately trained staff and standardized protocols. Developing interdisciplinary workflows and guidelines to support safe holding practices during TH, particularly for clinically stable infants and, when feasible, for selected infants with higher acuity, can enable more infants and their families to receive the benefits of holding during this critical period of neonatal care.

### Limitations

As a retrospective study, our ability to infer causality between holding and improved outcomes is limited by potential residual confounding. Infants who were held during TH were generally more clinically stable, and clinicians may have preferentially selected these infants for holding. Although our analyses adjusted for illness severity and respiratory support, unmeasured aspects of clinical stability and provider judgment may have influenced the observed associations.

Sedation practices and cooling device selection were not standardized across sites, and available data lacked granularity regarding timing, dosing, duration, and temperature stability during holding. Sedation may function as a proxy for illness severity, a mediator of care practices, or both. These factors may introduce residual confounding and limit causal interpretation.

Holding practices were not protocolized for most of the study period. At the end of the study period, one site began implementing a formal protocol to support parental holding during TH, likely reflecting a gradual transition in practice. Although adjustment for admission year and site was performed, residual confounding related to temporal changes in practice may persist. The study period also included the COVID-19 pandemic, during which visitation restrictions, limitations on parental presence, and infection control policies may have reduced opportunities for holding.

Holding data were derived from electronic medical record documentation and may not reflect the actual duration or quality of holding, nor identify which caregiver performed the holding. We were also unable to determine the specific reasons why infants were not held, including whether this was due to clinical factors, parental preference, parental presence in the NICU, or logistical and institutional barriers. This limits our ability to distinguish between medically appropriate non-holding and potentially modifiable barriers to family engagement. Additionally, we did not examine specific modes of respiratory support, which may further influence decisions around holding.

Finally, the study population included a substantial proportion of infants with mild encephalopathy, for whom the benefit of TH remains uncertain. This may limit generalizability to populations with more severe illness or to settings with different treatment practices.

Future prospective studies are needed to address these limitations by incorporating real-time measures of parental engagement, detailed characterization of clinical and contextual factors, qualitative data on caregiver and staff perspectives, and long-term neurodevelopmental outcomes. Despite these limitations, this study provides important preliminary evidence on the potential value of holding during TH, reinforcing the need to promote consistent, family-centered care in neonatal neurocritical settings.

## Supplementary Material

Supplemental Table 1

Supplemental Figure 1

**Supplementary information** The online version contains supplementary material available at https://doi.org/10.1038/s41372-026-02753-3.

## Figures and Tables

**Fig. 1:**
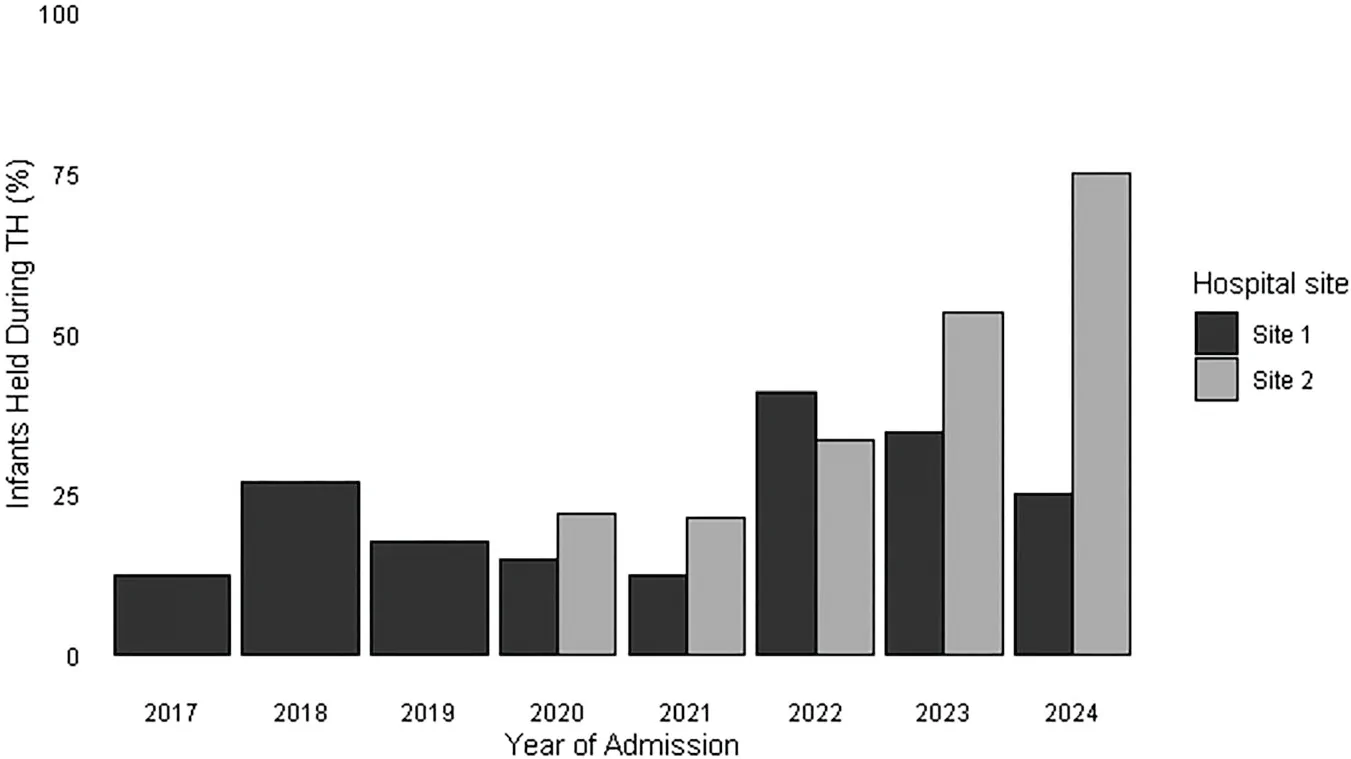
Percent of Infants Held During Therapeutic Hypothermia by Admission Year and Hospital Site.

**Fig. 2:**
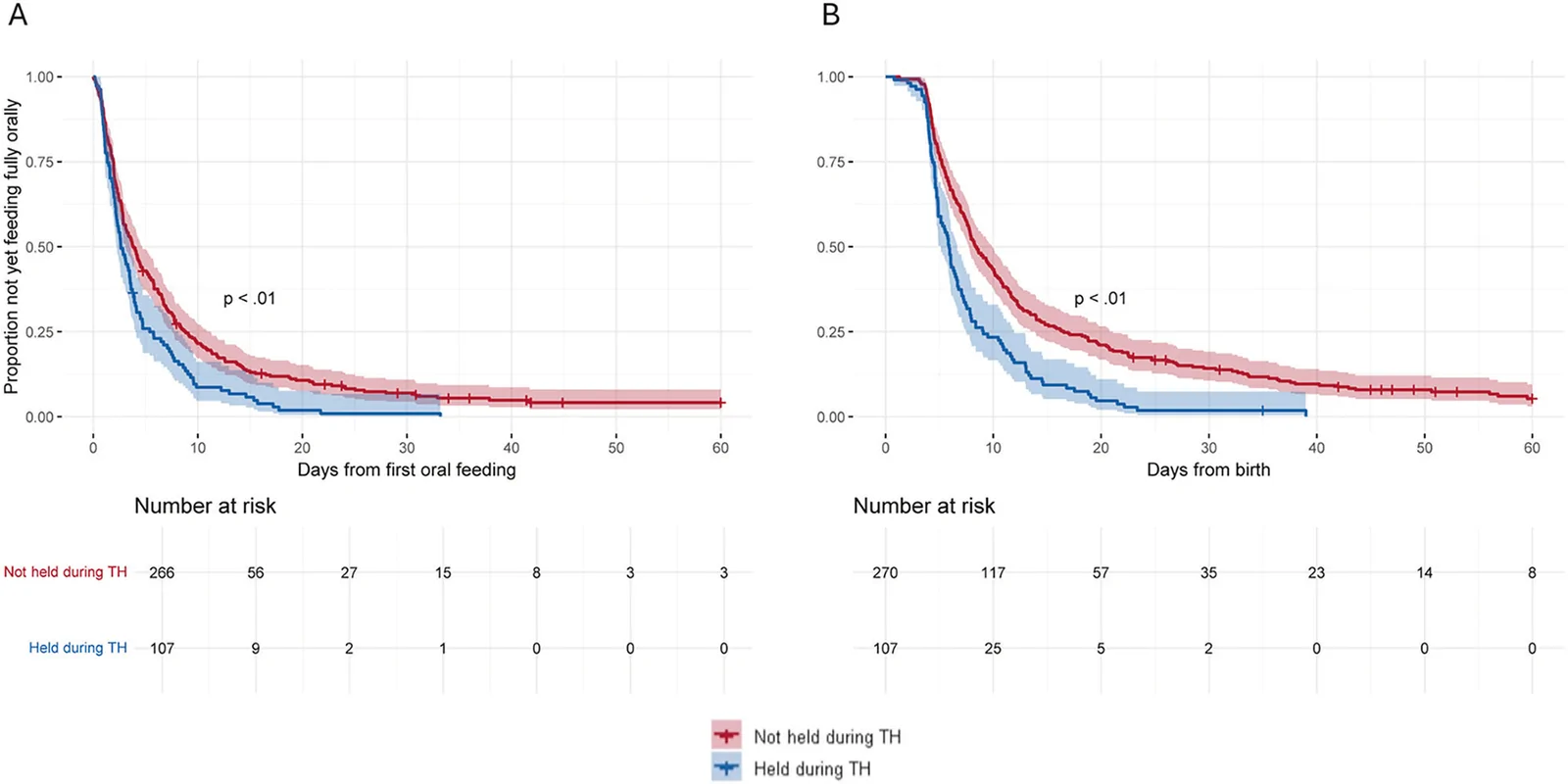
Kaplan-Meier Curves for Time to Full Oral Feeding by Holding During Therapeutic Hypothermia (TH). **A** Time to full oral feeding from first oral feeding (*n*= 373; 17 censored). **B** Day of life at full oral feeding (*n*= 377; 21 censored). Shaded bands show 95% CIs; crosses denote censored infants. Numbers at risk are shown below each panel. Log-rank tests compare groups.

**Table 1.** Comparison of Infant Characteristics and Holding Measures by Holding During Therapeutic Hypothermia.

	Held during therapeutic hypothermia (*n* = 107, 28%)	Not held during therapeutic hypothermia (*n* = 272, 72%)	*p*-value
**Infant Characteristics**			
Birth gestational age (weeks)^a^	39.5 ± 1.6	39.4 ± 1.6	0.58
Birth weight (kg)^a^	3.3 ± 0.6	3.3 ± 0.5	0.96
Male sex^b^	62 (58%)	151 (56%)	0.75
Infant race and ethnicity^b^			0.01*
White	46 (43%)	77 (28%)	
Hispanic	11 (10%)	38 (14%)	
Asian	7 (6%)	35 (13%)	
Black	2 (2%)	21 (8%)	
Multiracial	8 (8%)	12 (4%)	
Other	33 (31%)	89 (33%)	
Public insurance^b^	34 (32%)	153 (56%)	< 0.01*
Cesarean section^b^	48 (45%)	150 (55%)	0.09
1-minute Apgar score^c^	2 [1,3]	2 [1,3]	0.22
Unknown	2	5	
5-minute Apgar score^c^	5 [3,7]	4 [3,6]	0.03*
Unknown	2	6	
Severity of encephalopathy^b^			0.27
Mild	50 (47%)	122 (45%)	
Moderate	51 (48%)	120 (44%)	
Severe	6 (5%)	30 (11%)	
Arterial cord pH^a^	7.2 ± 0.2	7.2 ± 0.2	0.61
Arterial cord base excess^a^	−7.1 ± 5.7	−7.0 ± 6.3	0.85
MRI showing basal ganglia, thalamus, or brainstem involvement^b^	8 (8%)	37 (14%)	0.14
Seizures^b^	18 (17%)	66 (24%)	0.15
Continuous sedation medication^d^			
Morphine	32 (30%)	148 (55%)	
Dexmedetomidine	48 (45%)	60 (22%)	
Both	27 (25%)	61 (22%)	
None	0 (0%)	3 (1%)	
Number of PRN morphine^c^	2 [1,5]	2 [1,5]	0.95
History of intubation^b^	18 (17%)	127 (47%)	< 0.01*
Intubation days^a^	0.2 ± 0.6	1.0 ± 1.4	< 0.01*
Hospital site^b^			0.03*
Site 1	43 (40%)	145 (53%)	
Site 2	64 (60%)	127 (47%)	
Gastrostomy tube placement^d^	1 (1%)	18 (7%)	
**Holding Measures**			
Time to first hold (days)^c^	1.4 [0.9, 2.1]	4.0 [3.6, 4.9]	< 0.01*
Daily holding frequency^c^	3.0 [2.4, 3.9]	2.5 [1.8, 3.2]	< 0.01*

Categorical data presented as count (proportion of column total). Continuous data presented as mean ± standard deviation or median [Q1,Q3]. *MRI* magnetic resonance imaging, *PRN* pro re nata (as needed), *GT* gastrostomy tube, *NICU* neonatal intensive care unit.

a *p*-value from independent samples t-test.

b *p*-value from Pearson’s chi-square test.

c *p*-value from Wilcoxon rank-sum test.

d *p*-value not calculated given small representation across cells.

* *p* < 0.05.

**Table 2.** Associations Between Holding During Therapeutic Hypothermia and NICU Outcomes.

	Time to full oral feeding			Day of life at full oral feeding			Length of stay			Breastmilk at discharge		
	Median [Q1,Q3]	Unadjusted HR (95% CI)	Adjusted HR (95% CI)	Median [Q1,Q3]	Unadjusted HR (95% CI)	Adjusted HR (95% CI)	Median [Q1,Q3]	Unadjusted β (95% CI)	Adjusted β (95% CI)	*N* (%)	Unadjusted OR (95% CI)	Adjusted OR (95% CI)
Not held during TH	3.6 [1.8, 7.7]*	Ref.	Ref.	7.9 [5.0, 13.6]*	Ref.	Ref.	11 [8,20]*	Ref.	Ref.	211 (78%)*	Ref.	Ref.
Held during TH	2.6 [1.4, 5.2]*	1.5 (1.2, 1.9)*	1.4 (1.1, 1.9)*	5.9 [4.4, 8.8]*	1.9 (1.5, 2.4)*	1.6 (1.2, 2.0)*	8 [7,12]*	−7.5 (−11.4, −3.6)*	−4.9 (−9.1, −0.7)*	98 (92%)*	3.2 (1.6, 7.0)*	2.5 (1.1, 6.1)*

Adjusted models included severity of encephalopathy, number of intubation days, race/ethnicity, insurance type, admission year, and site. Cox proportional hazards models were used for time to full oral feeding and day of life at full oral feeding, linear regression for length of stay, and logistic regression for breastmilk feeding at discharge.

*HR* hazard ratio, *β* beta coefficient, *OR* odds ratio, *CI* confidence interval, *Ref* reference.

* *p* < 0.05

## Data Availability

The data used to support the findings of this study are available from the corresponding author upon reasonable request.
