# Supplementary material for: Association of parental holding during therapeutic hypothermia and NICU outcomes for infants with hypoxic-ischemic encephalopathy
Source: J Perinatol. Author manuscript; Available in PMC 2026 Nov 9. (PMC13747203; doi:10.1038/s41372-026-02753-3)
Supplement: Supplementary file 1 [file NIHMS2216033-supplement-Supplemental_Figure_1.docx]

**Supplemental Figure 1.** Conceptual Causal Diagram of the Relationship between Parental Holding during Therapeutic Hypothermia and NICU Outcomes
