# Supplementary material for: Association of parental holding during therapeutic hypothermia and NICU outcomes for infants with hypoxic-ischemic encephalopathy
Source: J Perinatol. Author manuscript; Available in PMC 2026 Nov 9. (PMC13747203; doi:10.1038/s41372-026-02753-3)
Supplement: Supplementary file 2 [file NIHMS2216033-supplement-Supplemental_Table_1.docx]

**Supplemental Table 1.** Sensitivity analyses excluding race/ethnicity and insurance from adjusted models

| Outcome | Sensitivity Model |
| --- | --- |
| Time to full oral feeding (HR) | 1.6 (1.2, 2.0)* |
| Day of life at full oral feeding (HR) | 1.8 (1.4, 2.3)* |
| Length of stay (β) | -6.6 (-10.6, -2.6)* |
| Breastmilk at discharge (OR) | 3.9 (1.8, 9.1)* |

Sensitivity model excludes race/ethnicity and insurance from adjusted analyses.

HR, hazard ratio; OR, odds ratio; β, mean difference in days; CI, confidence interval.

**p*<.05
